# Report From the 2022 Mary Tyler Moore Vision Initiative Diabetic Retinal Disease Clinical Endpoints Workshop

**DOI:** 10.1167/tvst.12.11.33

**Published:** 2023-11-28

**Authors:** S. Robert Levine, Martin G. Myers, Ryan Barunas, Dolly S. Chang, Sanjoy Dutta, Ted Maddess, Jeffrey M. Liebmann, Steve Sherman, Melvina Eydelman, Jennifer K. Sun, Wiley Chambers, Kerstin Wickström, Ulrich F. O. Luhmann, Martin Pallinat, Adam Glassman, Lloyd Paul Aiello, Dorene S. Markel, Thomas W. Gardner

**Affiliations:** 1 Mary Tyler Moore Vision Initiative, Greenwich, CT, USA; 2 University of Michigan Caswell Diabetes Institute, Ann Arbor, MI, USA; 3 JDRF Northeast Ohio & Michigan Chapter, Southfield, MI, USA; 4 Genentech, South San Francisco, CA, USA; 5 JDRF, New York, NY, USA; 6 John Curtin School of Medical Research, Australian National University, Canberra, Australia; 7 Edward S. Harkness Eye Institute, Columbia University Medical Center, New York, NY, USA; 8 Regeneron Pharmaceuticals, Tarrytown, NY, USA; 9 Food and Drug Administration, Rockville, MD, USA; 10 Joslin Diabetes Center, Harvard Medical School, Boston, MA, USA; 11 Food and Drug Administration, Silver Spring, MD, USA; 12 Icelandic Medicines Agency, Reykjavik, Iceland; 13 Roche Pharmaceutical Research and Early Development, Translational Medicine Ophthalmology, Roche Innovation Center, Basel, Switzerland; 14 Visiontree Software, Inc., San Diego, CA, USA; 15 Jaeb Center for Health Research, Tampa, FL, USA; 16 Department of Learning Health Sciences, University of Michigan, Ann Arbor, MI, USA; 17 Department of Ophthalmology & Visual Sciences, Kellogg Eye Center, University of Michigan, Ann Arbor, MI, USA

**Keywords:** diabetic retinopathy, diabetic retinal disease, neurovascular unit, clinical endpoints

## Abstract

The Mary Tyler Moore Vision Initiative Diabetic Retinal Disease (DRD) Clinical Endpoints Workshop was held on October 22, 2022 to accelerate progress toward establishment of useful clinical and research endpoints and development of new therapeutics that have important relevance across the full spectrum of DRD pathology. More than 90 patient representatives, clinicians, scientists, funding and regulatory agencies, diagnostic, therapeutic and biotech industry representatives discussed the needs for new diagnostic and therapeutic approaches to prevent and restore retinal neurovascular unit integrity. Phase I of the MTM Vision Initiative plans, notably updating the DRD staging system and severity scale, establishing a human ocular biorepository and resource, and clinical endpoints and biomarker development and validation, was emphasized.

## Introduction

Diabetic retinopathy, now termed “diabetic retinal disease” (DRD), impacts the entire neurovascular retina^1^ and affects 103 million persons worldwide^2^ despite widespread availability to laser and anti-vascular endothelial growth factor (VEGF) treatments. Standard systems that assess disease severity are based on decades old photographic grading scales that reveal only the vascular lesions, does not include the midperipheral retina, and are not quantitative constrain the diagnosis and treatment of early stage disease, and are do not reveal the mechanisms of vision impairment in persons with DRD.^3^ Moreover, the pathophysiological basis of human DRD remains poorly understood owing to a lack of studies of human tissues. Thus, new efforts are need to advance this aspect of the field.

The inaugural Mary Tyler Moore Vision Initiative (MTM Vision) Diabetic Retinal Disease Clinical Endpoints Workshop was held in Ann Arbor, Michigan, on October, 25, 2022, and attended by 77 persons from 55 organizations, including patients and their representatives, clinicians, vision scientists, pharmaceutical and diagnostic device companies, and regulatory agencies (Supplementary Table S1). Platform presentations and breakout sessions addressed the imperative for an updated grading scale, the regulatory framework in which it would be developed, and the role of four areas: visual function and retinal physiology endpoints; patient-reported outcomes (PROs); systemic, biochemical, and systemic biomarkers; and retinal imaging. There was a very strong consensus that cooperative efforts across all aspects of DRD in a precompetitive environment was essential to move the field forward and to accomplish the goal of eliminating vision loss from diabetes (marytylermoore.org).

Dorene Markel, Managing Director of the MTM Vision, welcomed the participants and described the background of how the Initiative grew from an idea inspired by Mary Tyler Moore's vision loss owing to type 1 diabetes, and has blossomed into an international group of patients, scientists, clinicians, industry and regulatory experts who wish to overcome the current limitations.

Dr. Martin Myers, Director of the University of Michigan Caswell Diabetes Institute, described the partnership with the MTM Initiative. DRD remains an enormous world-wide problem despite advances in anti-vascular endothelial cell growth factor therapy, continuous glucose monitors, and insulin pumps. Dr. Myers also indicated that one of the major tasks for the Caswell Diabetes Institute is to identify needs within our community and convene groups of people around these needs with the idea that together we can do more than we can as individuals. He argued this proposition should be true for those at this meeting. He emphasized it will take a lot of directed thought and focus on everyone's part to get this to where it needs to be at the end of the day, and that is just the beginning of the process.

## S. Robert Levine

**Figure 1.**
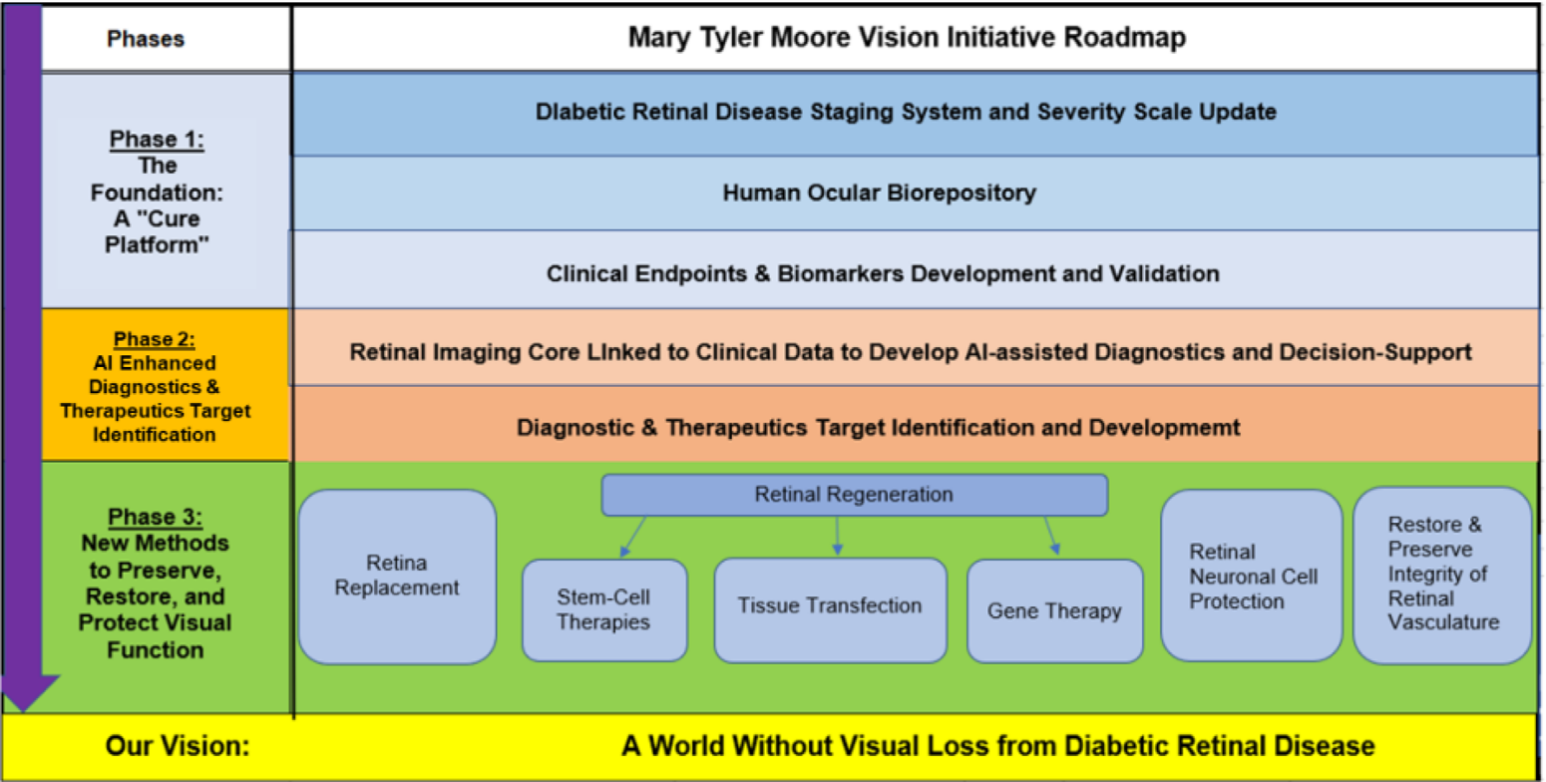
The MTM Vision Roadmap with three phases, with the goal to create a world without vision loss from DRD.

Dr. S. Robert Levine, the concept creator and chair of the MTM Vision offered welcoming remarks. Dr. Levine is a cardiologist, who, in the late nineteen eighties, stepped away from his faculty position in cardiology at the Mt. Sinai Medical Center in New York to join his wife, Mary Tyler Moore, in leadership of the Juvenile Diabetes Research Foundation (JDRF) and efforts that they made, together, to help find cures for type 1 diabetes and its complications. Dr. Levine put the Clinical Endpoints Workshop in the context of the broader MTM Vision (Fig. 1).

Diabetes had a devastating impact on Ms. Moore, in particular owing to her visual loss from DRD and the severe narrowing of her visual fields from panretinal photocoagulation therapy. MTM Vision was launched to honor her and her efforts to help find cures for diabetes and its complications, and importantly to focus attention on vision in its multiple meanings to people like her. That is, the vision that has been lost by millions of people with diabetes related eye disease; the vision that will be lost despite the advanced treatments available for diabetic rental disease; and the vision required to imagine a cure for vision loss, and to take those specific actions needed to find the ways to restore and preserve vision in people with diabetes and protect the retina from the deleterious effects of diabetes. The Clinical Endpoints Workshop is an important part of achieving these person-centered goals.

The mission of MTM Vision is to lead in accelerating the development of methods to preserve and restore visual function in people with diabetes including, those who already have significant visual loss for which there is an unmet need as evidenced by Ms. Moore's experience and the loss of vision in millions of others. MTM Vision's approach is to act as a catalyst by providing globally accessible critical-path resources, and leading convenings like the Clinical Endpoints Workshop, along with creating links to collaborative research networks who are willing to share their data in real time.

MTM Vision's roadmap describes its phase one as an accelerator that responds to three fundamental barriers to accelerated progress. First, you cannot solve a problem you have not defined. Second, you cannot find cures for human disease without studying the human condition. And third, you cannot judge success unless you know what to measure.

The response to the first challenge, “You cannot solve a problem you have not defined,” is MTM Vision's ongoing DRD staging update project, in which many attendees of the workshop participate and which informs MTM Vision's work on clinical endpoints. MTM Vision will lead development of an updated multidimensional DRD staging system which incorporates measures of visual function and retinal physiology, the molecular milieu—whether by blood samples, aqueous or vitreous humor evaluation—systemic factors, and, importantly, bringing the patient voice into the picture to better diagnose the disease earlier, better assess severity and risk of progression, better evaluate response to therapy, and better support clinical trials and therapeutics development. A natural output of the DRD staging update process is the identification of promising biomarkers and clinical endpoints that need further development and validation.

MTM Vision's response to the second challenge, “You cannot find cures for human disease without studying the human condition,” is standing up an ocular biorepository at the University of Michigan under the direction of Dr. Patrice Fort. The MTM Vision biorepository is modeled on the highly successful JDRF Network for Pancreatic Organ Donors with Diabetes (jdrf.nPOD.org), which has changed the way in which types 1 and 2 diabetes in humans are understood. The MTM Vision biorepository collects and characterizes cadaveric samples under a special protocol developed by Dr. Fort to ensure sample suitability for completing the detailed -omic analysis needed to better understand the cellular and molecular markers associated with progression of diabetic rental disease and identify therapeutic targets. This information will be put on a sample and data sharing platform available to the community and form the basis for a precompetitive industry consortium to accelerate therapeutics development. Dr. Fort has acquired a unique imaging tool (OCX; LighTopTech, Rochester, NY) which allows simultaneous acquisition of color photographs and optical coherence tomography (OCT) of the retina to better understand the disease and isolate segments for sampling and deep characterization.

The Clinical Endpoints Workshop has been designed to address the third challenge, “You cannot judge success unless you know what to measure.” The principal purpose of the workshop and one of the main goals of the consortium that will follow, is to identify promising clinical endpoints, including biomarkers, imaging methods, functional assessments, and PROs that have potential to become registrable endpoints, and plan trials that are necessary to learn more about potential for those endpoints.

Looking at the other two phases of the roadmap (Fig. 1), MTM Vision moves from these fundamental accelerators noted above to development of advanced diagnostics and therapeutics target identification. Included in this phase 2 will be a retinal imaging core, which will bring together millions of images associated with longitudinal clinical records and functional assessment to try and get some signal out of it using artificial intelligence tools for better diagnosis and prediction of course. Additionally, MTM Vision's phase 2 will model the successful Michigan Kidney Translation Core by developing a therapeutics target identification core to serve the global academic community and needs of precision medicine while establishing a critical resource and additional value for our precompetitive consortium of industry leaders, as well. Completing the goals of MTM Vision's phase 1: (a) DRD Staging Update project; (b) Ocular Biorepository; and (c) Clinical Endpoints Identification and Validation projects will potentially lead to (a) new indications for treatment in DRD, including earlier stage disease; (b) new targets at the molecular and cellular level for therapeutics development; and (c) new regulatory pathways for assessment of safety, efficacy, and, ultimately, new approaches to restore and preserve vision in people with diabetes, thereby de-risking the space, and, hopefully, incentivizing investment in DRD indications.

## Ryan Baranus

Mr. Barunas described his personal journey with diabetic retinopathy, including detached retinas in both eyes. He asked for a show of hands of persons who in the audience who have type 1 diabetes or has a loved one with type 1, and many people so acknowledged.

Ryan went for his annual eye examination in 2017, at which time he had no symptoms, no reasons for concern. He was shocked when was told that the doctor had discovered that I had swelling in the blood vessels in his left eye at the age of 31, a shock to him and his family. Two days later he received his first aflibercept eye injection, followed by scatter laser therapy. Despite these treatments he lost peripheral vision owing to a detached retina and required a vitrectomy and more laser therapy. It was a scary experience, and something that he still has nightmares about to this day. Two years later he woke up with blurred vision in his right eye owing to another detached retina. He was fortunate to have the great care of my doctors, so it was a good outcome for him, but it is not the same for everyone. He has a close friend through the JDRF who went through the experience just a few years earlier and who limited his career, does not have a driver's license, and to this day he still deals with double vision and blurred vision. He came through the ordeal but is concerned what the future will hold. Type 1 diabetes can cause an even greater emotional toll that it does a physical manifestation. Sometimes he is overwhelmed about the potential for reoccurrence, with the potential consequences that would have on his quality of life, his independence, career, and someday seeing his children achieve milestones in their lives.

Ryan meets a lot of families with young kids who have type 1 diabetes and is asked for words of advice for so many, some encouragement. He tells them that with technology with such as closed loop systems, good carb-counting skills, and proper diet and exercise, they will live a pretty normal life. Sometimes he shares that the constant battle to control type 1 diabetes will create a mental fortitude that will benefit them in everything they do. The unfortunate reality is that, as resilient as we are and as our bodies can be, diabetes is a terrible and unrelenting disease. Diabetics are all at risk of complications, and he believes the most recent statistics show that 35% of persons with type 1 diabetes will develop DRD at some point in their lives.

Ryan and his fellow diabetics and completely rely on our hard work and research. He asks us to achieve earlier diagnosis, more effective early-stage treatment, and ability to recover lost vision so that he and others like him can regain their independence, and so that when we meet a newly diagnosed child, we can look them in the eye and tell them that they have a bright future.

## Dolly Chang

The medical community recognizes the transformative impact of intravitreal anti-VEGF over the last decade. However, concerning diabetic retinopathy, a challenging path lies ahead. Dr. Chang draws attention to unmet needs in drug development and underscores the significance of clinical endpoints.

### Therapeutics

Multiple clinical trials have demonstrated that anti-VEGF therapy can improve the Diabetic Retinopathy Severity Score (DRSS) and reduce the risk of developing vision-threatening complications.^4^^–^^6^ However, only about a quarter of retina specialists consider its use for patients with severe nonproliferative diabetic retinopathy (NPDR) without diabetic macular edema.^7^ Dr. Chang suggests this limited adoption might stem from uncertainties regarding anti-VEGF's role as a true disease-modifying therapy. Untreated patients with mild DRD tend to progress at a slow rate. Yet, those with mild disease severity owing to anti-VEGF treatment tend to experience more rapid progression once treatment is discontinued.^8^ Furthermore, anti-VEGF might not induce retinal reperfusion, a primary factor in the progression of DRD.^9^

Evidence suggests that patients with diabetes may begin to experience retinal neurodegeneration before microvascular changes are clinically evident.^10^ Over time, these individuals are at a two- to three-fold increased risk of retinal ganglion cell loss compared with age-matched controls. When assessing retinal sensitivity using microperimetry, a decline is already evident in patients with no or mild DR.^11^ Moreover, the 5-year follow-up of patients in the Protocol S clinical trial showed continued visual field degradation after receiving either panretinal photocoagulation or ranibizumab.^12^ Given that VEGF is vital for retinal cell survival, it is doubtful that anti-VEGF treatment by itself can halt or reverse the neurodegeneration process.

Reviewing clinicaltrials.gov, Dr. Chang notes multiple active industry-sponsored clinical trials, primarily focused on inflammation or the VEGF pathway. This finding points to the necessity for therapeutic strategies extending beyond VEGF and inflammation, potentially delving into neuroprotection or reperfusion.

### Clinical Endpoints

Although the two-step DRSS improvement is an established endpoint, Dr. Chang believes it might constrain the development of novel non–anti-VEGF molecules. The DRSS may not fully capture the extent of neurodegeneration or perfusion status. Importantly, an improved DRSS might not always equate to tangible visual benefits. Dr. Chang advocates for innovative structural and functional endpoints to bridge the gap.

Given these concerns, there is a pressing need to develop new clinical endpoints. Throughout the drug development process—from early proof of activity to proof of concept, and through to pivotal trials—refining these endpoints is crucial. Initially, this involves quantifying structural changes, such as nonperfusion areas or regions with retinal mitochondrial stress. It is essential to interpret the functional implications of these structural alternations. For example, how do nonperfused areas impact retinal sensitivity? In the final stages of trials, it becomes critical to demonstrate that any therapeutic effects directly benefit the patient's vision.

### Patient Selection

Dr. Chang notes the prevalent trend of anti-VEGF trials enrolling patients with a DRSS of 47 to 53. Demonstrating treatment benefits for those with earlier or later stages is challenging. The progression is slow in early stages, and it might be ethically questionable to use placebos for advanced cases. For non-anti-VEGF trials, reliance on DRSS seems inadequate, underscoring the need for natural history studies. Dr. Chang emphasized the imperative to embrace new technologies to characterize disease pathology comprehensively. Such advances could streamline trial designs, enabling the discernment of significant treatment benefits within a more concise timeframe.

## Sanjoy Dutta

Dr. Dutta is the Chief Scientific Officer at JDRF where their mission is to accelerate the development of life-changing breakthroughs that treat, prevent and cure type 1 diabetes and its complications, while the vision is a world without type 1 diabetes. Dr. Marjana Marinac from the JDRF regulatory policy team also participated in the workshop. JDRF supports research and advocacy throughout the world to find treatments and cures for type 1 diabetes, and they are the world leading funder of type 1 diabetes research, but they cannot do it alone. Now have we been able to make progress so far, and how we are aware of where we need to go?

The consortium approach bring multidisciplinary approaches together to solve complex problems, which are not going to be solved by any individual. One of the seminal discoveries JDRF led with many investigators, other funding organizations, and the regulators is staging type 1 diabetes.^13^ When type 1 diabetes is diagnosed in an individual, it is with an artificial cut-off value of hemoglobin A_1C_ levels and then a person is put on insulin treatment when there is insufficient insulin production. We found through more than 30 years of research knowledge, natural history of pathogenesis, and symptomatology in the disease stages before one becomes insulin dependent, that one has the disease years, maybe decades before one is diagnosed with a specific hemoglobin A_1C_ value and put on insulin treatment. Why do we not study those individuals? Prevention can be much easier than cures in many cases. Why can not we have preventive strategies? Once we could stage type 1 diabetes and this became the new roadmap to follow, and regulators and payers had buy-in, it immediately opened the floodgates for research and development for therapies to address the disease at early stages.

The diagnosis is now labeled as stage three diabetes. Before the revised staging paradigm a person was diagnosed with type 1 diabetes when they were hyperglycemic, so it was not called stage one, but now this is stage three. There's a stage zero, one, and two, but that predates insulin dependence by years or decades. This is clinically and biologically where you can actually have measures, critical endpoints about beta cell function, insulin production, about autoantibody presence in the blood. And now, with increasing evidence from continuous glucose monitoring about the glucose imbalances in real time that are not picked up by a hemoglobin A_1C_ number, is a key advance that has now gotten the research community to find preventive therapies and disease modifying therapies when type 1 diabetes has just been diagnosed.

Another example of where this approach has been helpful is clinical endpoints. Hemoglobin A_1C_ is a very important number. Through the DCCT (1983–1993) and other follow-up studies, it is one of the best surrogates for predicting long-term complications. However, we know it is not reflective of the day-to-day life diabetes management challenges of people with diabetes, so there are outcomes beyond A_1C_ that warrant consideration: time in different glucose ranges, hyperglycemia, and diabetic ketoacidosis that have incredible value to the person with diabetes, and are clinically meaningful.

These outcomes have been incorporated by regulators for approval of devices. The JDRF works hard with regulatory agencies in and outside the United States to ensure they are adopted for disease modifying therapies as well as novel future generation cell replacement therapies. The same holds true for other endpoints such a C-peptide and islet autoantibodies that were qualified by the European Medicines Association recently.

Second, this work opens translational pathways to intervene at various stages of the disease to arrest further progression. We work in partnership with the European Consortium in their Innovative Medicines Initiative, a public–private partnership, that addresses gaps in precompetitive consortia. Similar approaches are taken with the JDRF's artificial pancreas consortium and stem cell-based insulin replacement therapy. It allows collaborations, partnerships, and reduces redundancies through a consortium approach. The artificial pancreas or automated insulin delivery system, has three components and, hence, requires a synchronized and deliberate collaboration. An artificial pancreas has the insulin-delivering pump, continuous glucose monitor, and an algorithm that controls the glucose value-based insulin delivery. Likewise, in a beta stem cell replacement therapy product, one has the stem cells, immune protection strategies, and important surgical transplantation procedures for graft survival with probably some local immunosuppression. These multicomponent products often require more than one company to work together. Challenges include research and development required to develop safe and efficacious product prototypes, multiple industry partners with individual components to work together toward a composite product, and the business case to ensure success for all parties. Outcomes beyond A_1C_ led to a consensus statement that is now being adopted by top leaders in the field.

The consensus statement was an important success in 2011 or 2012, with the JDRF leading the way, which led to the guidance document from the U.S. Food and Drug Administration (FDA) on clinical pathway to develop artificial pancreas therapies. This progress has paved the policy, product development and reimbursement strategies in the United States and around the world. Therefore, multiparty, multidisciplinary stakeholders, and engagement and commitment is only the way to go. But we must remember the center of the universe—that is, a person with diabetes. So, let's have a paradigm shift in the way we think, not only for collaborating and partnering, but also for the greater goal in mind. This lesson became clear early on in my career: bring the person with diabetes to the trial; do not bring the trial to the person with diabetes. So, a call to action: there is an urgent need to incorporate the person with diabetes perspective in product development and research and development.

## Tom Gardner

Dr. Gardner emphasized the success of the MTM Initiative relies on the contributions of many parties, and there are two broad questions at hand. The first is to test the hypothesis that incorporation of patient symptoms or other measures of retinal function can improve the diagnosis and treatment of DRD. There are reasonable preliminary data to support the hypothesis, but they must be tested rigorously. Medical students learn that the constellation of symptoms, physical signs, and diagnostic tests form the basis for evaluating all patients. Hence, determination of structure and function is important in all aspects of clinical medicine, including for complications of diabetes, notably cardiovascular disease or renal insufficiency. Moreover, assessment of function has always been an integral part of the assessment of patients with glaucoma and inherited retinal diseases. Diabetic retinopathy has not conventionally included that approach, but we can learn from these other fields and to better help patients.

The second question is can we, and will we, commit to working together to solve this problem? It is easy to say “yes” now, but this venture is at least a 5- to 10-year commitment. That means a lot of our lives, it means a lot of money, and a lot of collaboration. And why should we do this? Mr. Barrunas told us he did everything he was supposed to do as a patient, and yet he still has suffered. He had to have surgery and destructive laser because we are treating retinopathy at end stage—retinal failure. Our current approach is equivalent to waiting for diabetic kidney disease to progress to the point of requiring dialysis or renal transplantation—renal failure. If the current system of DRD diagnosis and treatment worked well persons like Ryan would have good vision. But our current system is not satisfactory, so will we do this? If, as Dr. Dutta stated, our emphasis is on the patients, so we must.

## Ted Maddess

Dr. Maddess listed five key criteria to determine useful structural and functional measures for endpoints:

1. Standardized effect sizes to discriminate controls from persons with diabetes but no DRD. Values of 2 or better are needed, corresponding to area under receiver operator characteristic curves of more than 0.9.
2. To assess independent dimensions of DRD, different measures should be as uncorrelated with each other as possible, but correlated with diabetes.
3. High reproducibility to track change over time.
4. Good correlation of measures with complications screening variables.
5. Spatially matched structure and function measures.

**Figure 2.**
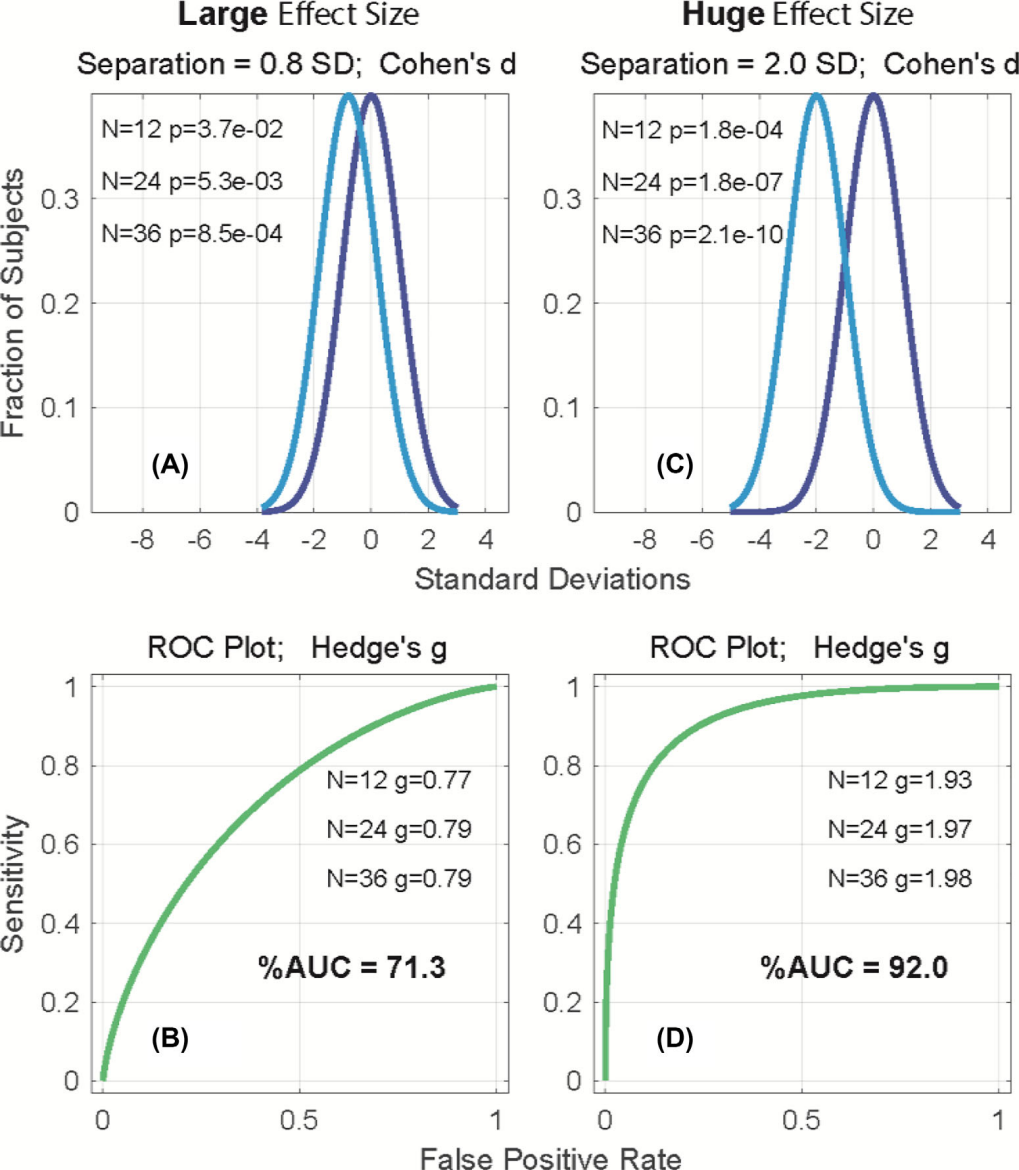
Examples of large and huge effect sizes as shown using Cohen's d and Hedge's g methods.

On criterion 1, Figure 2 illustrates the relationship between effect sizes, areas under receiver operator characteristic plots, and *P* values. Figure 2C shows examples of distributions of patient (blue) and control (purple) data. The distributions are 1 standard deviation (SD) wide, so their separation is equal to Cohen's *d*: the most common measure of effect size. Below each pair of distributions are the receiver operator characteristic plots defined by those distributions, indicating the percent area under the curve (AUC), showing that the effect size and the AUC are measures of diagnostic power. Figures 2A, and B show examples for a *large* effect size of 0.8, and a huge effect size of 2.0. The corresponding AUCs are 71.3% and 92.0%, respectively.

Figures 2A, and C show three rows of numbers with the *P* values for *n*s of 12, 24, and 36. The *P* values change hugely with the *n*. *P* values are meaningless in the clinic because you only have one patient; the only thing that matters is the relative separation of the distributions. Figures 2B, and D also have three rows of numbers—Hedge's *g*, which is Cohen's *d* corrected for sample size. The same *n*s are used as for the *P* values. Hedge's *g* changes little with the *n* and can be calculated from the means, SD, and *n*. This statistic can be useful to compare the clinical value of published measures. Note that, for Figure 2B at a sensitivity of 90%, we will misdiagnose three-quarters of the normal population. By comparison, using a huge effect size (Fig. 2D), we still misdiagnose one-quarter of the normal population. Effect sizes of 2 are the minimum.

Dr. Maddess used published data on the means, SD, and *n*, for different functional and structural tests to calculate Hedge's *g* and converted them to AUCs. He presented data for 14 measures from 7 functional tests. The median ± SD Hedge's *g* and AUCs were 0.40 ± 0.39 and 61.5 ± 10%. For 8 measures from 4 structural tests the values were 0.52 ± 0.25 and 65.0 ± 6.70%. For 9 measures from the Objective Field Analyzer being developed by Dr. Maddess, the values were 1.74 ± 0.34% and 89.0 ± 4.52%.

On criterion 2, uncorrelated measures, they should correlate with diabetic retinal damage, but any disease is multifactorial, and so measures that are less correlated with each other can characterize different disease aspects or phases. This is related mathematically to the likelihood ratio. The less correlated measures are with each other, the larger their combined likelihood ratio.

Criterion 3 is good reproducibility. Standard automated perimetry has poor test–retest variability, which limits our capacity to track change over time. DR is distributed across the retina so we should use some form of perimetry in diabetes.

Criterion 4 is correlation with independent factors within the standard complication screening variables. Dr. Maddess’ group has reported that the standard variables like body mass index, hemoglobin A_1C_, and estimated glomerular filtration rate contain about three independent factors. We can use this to our advantage for machine learning.

Criterion 5 is about matching structure and function. The parts of the retina that are sampled by these methods are completely different. If we measure structure and function in matched parts of the retina, then we can Criterion 2 to increase our diagnostic power. Dr. Maddess presented data from an Objective Field Analyzer perimetry test, M18, whose stimuli match the Early Treatment Diabetic Retinopathy Study (ETDRS) grid used by all OCTs to report macular thickness data. Thus, structure and function can be compared at the same retinal regions. M18 tests both eyes in less than 90 seconds and each M18 region produces a response sensitivity and a delay.

The audience queried what was the best criterion for early stage diabetic eye damage, hemoglobin A_1C_, or duration of disease, or both? Dr. Maddess emphasized that compared with other eye diseases, one often has a good idea when diabetes starts. Dr. Rafael Simó asked if Objective Field Analyzer is influenced by cognitive impairment? Dr. Maddess said that his group have worked to reduce attentional effects. They have published on conclusions and have begun work on Alzheimer's. He supposed that cognitive impairment is an issue in later stage diabetes.

## Jeffrey Liebmann

Many of the ideas under discussion today are paralleled in the field of glaucoma and one could substitute the word glaucoma with DRD. Developing structural and functional endpoints for glaucoma neuroprotection clinical trials are vital if we are to develop new interventions for the disease.

In glaucoma, we have data from longitudinal studies, including the Ocular Hypertension Treatment Study Collaborative Initial Glaucoma Treatments study, among others. The Ocular Hypertension Treatment Study randomized patients with ocular hypertension, without evidence to glaucoma, to treatment (intraocular pressure lowering) and untreated arms. The Memantine and Low-Pressure Glaucoma Treatment studies were clinical studies of neuroprotection. There is also regulatory guidance from the FDA regarding potential glaucoma visual function endpoints. The most recent pressure lowering randomized trial, the United Kingdom Glaucoma Treatment Study, followed 250 patients for several years, and there was a clear difference between the treated and untreated arms at two years. Separation between the groups occurred within 1 year.

In the Low-pressure Glaucoma Treatment Study, with roughly 150 patients, we demonstrated a difference between two accepted treatments—timolol and brimonidine. Even if the actual mechanism here was not purely neuroprotection, we demonstrated separation between the two groups with relatively few patients.

One problem in the field of glaucoma is the relative lack of published, approved regulatory endpoints. The glaucoma community has attempted to solve this problem by getting all stakeholders into the same room and working together to create some common ground for the future discussion. Two published articles led by Drs. Bob Weinreb and Paul Kaufman brought together the FDA, the National Institutes of Health, ARVO, and the entire glaucoma community to look at possible strategies.

We recently published a trial on the effect of nicotinamide on neuro-recovery in glaucoma patients.^14^ We used visual field clustering, a relatively new concept, doing four visual fields at baseline and follow-up, to look for treatment effects. We have not yet demonstrated the correlation between OCT structural change and visual field change, but an agreed-upon structural endpoint would be a great advance for glaucoma clinical trials.

Many members of the audience are familiar with the basic science that came out of Dr. Simon John's laboratory in *Science* in 2017. Our vitamin B_3_ study was randomized and prospective, enrolling 32 patients. In 2 months with clustered visual fields, we generated an uptick in sensitivity and statistical difference between the groups in the number of points in the field demonstrating some improvement. This means that the drug actually got to the target tissue and had some effect on that target tissue using this novel testing strategy. The result is similar to the photopic negative response by Dr. Johnny Crowston's group in Australia using nicotinamide alone. There are now five trials worldwide looking at nicotinamide in glaucoma using phase 3 designs

The Memantine trial visual field endpoint was defined as five or more reproducible points using change probability analysis. The study enrolled 2000 patients worldwide and took four years. This is an unacceptably high number of patients and the duration is too long. Dr. Liebmann feels most companies could support a 2-year trial, but would need to limit the number of patients. Designing phase 2 trials is also a huge hurdle, because we do not have an ideal surrogate, which we are still searching for.

Dr. Liebman responded to a question regarding how can we link structural changes to a clinically significant change in function that would be acceptable to regulators? That linkage is missing owing to difficulties from a regulatory perspective. The OCT structural measure is more sensitive, more reproducible, more reliable, and more repeatable than perimetric change, but is a small change of one micron in OCT thickness meaningful for a patient or a regulator?

## Steve Sherman

Anti-VEGFs and PRP improve visual acuity in patients with PDR, but few qualitative research studies describe the treatment effects from a patient perspective. PROs are a good way to gain insights into patient function and well-being following treatment, but to date no content valid PRO instrument has evaluated the effects of treatment on symptoms, functional impacts, and health related quality of life in patients with DR.

This study developed a de novo PRO instrument to assess symptoms, impacts, and health related quality of life in patients with PDR who are receiving treatment. A literature review identified qualitative research that describes the patient experience with DR. Three clinicians interviews enhanced concept elicitation and identified additional feedback on concepts in the literature review. Existing PRO instruments used in PDR and other ophthalmic conditions were identified, and key ones that we felt relevant to map against the preliminary conceptual model of disease were selected. A few rounds of patient interviews further refined the PRO instrument and our conceptual model of diabetic retinopathy.

Eighty-four concepts from PubMed were identified from 1754 articles and 22 concepts were identified. Four websites were sourced, including a patient organization, medical organizations, and online forums, and three concepts from the clinician interviews were identified. Together, 106 concepts were included in our preliminary conceptual model.

Twelve PRO instruments were identified from PubMed with a review of 409 abstracts, 10 PRO instruments from clinical trials from review of 363 records, and an additional PRO through manual search. Of those, five instruments were selected, which included the NEI-VFQ-25, the impact of visual impairment questionnaire, the retinopathy dependent quality of life questionnaire, the limited luminescence questionnaire, and the RetCAT. These were mapped against our preliminary conceptual model. The RetCAT demonstrated the greatest conceptual coverage across symptoms and impacts, but did not adequately cover concepts related to the treatment experience. Hence, we our diabetic retinopathy scale included four domains, including daily activities, emotional impacts, social impacts, and vision problems.

Demographics and clinical characteristics of our interview patients for round one and round two patients were mostly consistent. The mean age was 53 years, most were Black or African American, and the mean years since PDR diagnosis ranged from 6 to 8 years. In round one, 40 patients were interviewed, and 30 in round two.

The preliminary scale was revised iteratively based on patient feedback. Round one patients reported that instructions, items, and response options of the DR scale were well understood, but proposed modifications to reduce the redundancy and improve clarity in interpretation and relevance. The questionnaire was revised to include four new items in the daily activity scale, one new item in the emotional impact scale, and a new five-item treatment experience scale was added. The social impact scale was excluded because of irrelevance owing to the coronavirus disease 2019 pandemic. Based on the round two interviews, additional modifications improved the clarity and the interpretation. The final scale included 85 items and spanned four scales including daily activities, emotional impacts, vision problems, treatment, and in treatment experience.

Our final conceptual model included three domains including symptoms, impacts, and managing the disease. Red vision and dizziness were not previously associated with PDR, but were reported by our patients. A variety of eye problems were reported by our patients that were not previously reported. Several concepts were identified in the literature but did not come out of patient interviews; for example, handling cash, feeling like a burden, and decreased socializing. Concepts from the patient interviews were added, such as walking, writing, concerns for future worsening of symptoms, and loss of independence. The managing disease domain highlights the complexity of managing these patients. For treatment effects, a broad range of concepts emerged from the patients including worsening vision or eye problems, no change, or improved eye problems and vision.

In conclusion, a robust, comprehensive PDR-specific PRO was developed to evaluate symptoms, impacts, and health related quality of life in patients with PDR who are receiving treatment. The DR scale demonstrates sufficient granularity to evaluate clinical and functional benefit of PDR therapies, and the treatment experience scale was a unique component that assesses the experience of patients receiving treatment for PDR. Further refinement of the measure is warranted through a standalone study to confirm the scoring, and to reduce the number of items and to evaluate secondary properties.

## Melvina Eydelman

Dr. Eydelman works for the FDA Center for Devices and Radiological Health (CDRH), where patients are at the heart of all we do, and our principle is that patient input is valuable throughout the total product life cycle of a medical device, from discovery and ideation to clinical testing and on to post-market monitoring. Patient experience data includes clinical outcome assessments of four types which provide subjective information about how a patient feels, functions, or survives. It is important to note that they differ by the reporter. They are: performance outcome measures, clinician reported outcome measures, observer reported outcome measures, and PRO measures.

The roadmap for selection and development of the appropriate clinical outcome assessments is the same for all four. The underlying core principle governing all clinical outcome assessments is structured data collection. Reports of the status of a patient's health condition come directly from the patient without interpretation of the patient's response by a clinician or anyone else.

It is imperative to follow a structured approach in developing and administering PRO measures. To better communicate the FDA's thinking on this subject, a guidance was issued in 2009 that applies to all the medical product centers. It provides recommendations about the evidence used to support a PRO instrument-based labeling claims. To further the agency's efforts to include the patient experience data, the FDA committed to drafting new guidance documents that clearly spell out the regulatory perspectives on the development and use of clinical outcome assessments. Patient-focused drug development guidance was issued in June, of 2022. The CDRH published the final guidance principles for selecting, developing, modifying, and adapting PRO instruments for use in medical device evaluation. The guidance applies to PRO instruments used across the total product life cycle and is meant to supplement existing guidance and clarify whether there are areas of flexibility for medical devices. There are few best practices described in this guidance document that could be implemented to make the process of selecting, using, modifying, or adopting a PRO instrument for specific use within a clinical study more efficient. Collaboration allows for the pooling of resources financially and in terms of access to expertise and key stakeholders.

The FDA has participated in multiple collaborative efforts with all stakeholders in the medical device ecosystem. These collaborations led to completion of PRO measures for refractive surgery, intraocular lenses (IOLs), and glaucoma. Currently, we are in the early development stages of PROM for patients with profound visual loss.

Approximately 15 years ago, CDRH received complaints from patients regarding symptoms such as dry eyes, glare, halos, starburst, and double vision significantly affecting patient's quality of life following LASIK. To better understand the potential risk of severe problems that can result from LASIK, we partnered with the NEI and the Department of Defense to launch the LASIK Quality of Life Collaboration project. This study produced a questionnaire, the Patient Reported Outcomes with LASIK (PROWL). The questionnaire evaluates visual symptoms and satisfaction with vision following surgery and incorporates images and definitions to facilitate reporting of complex visual symptoms. PROWL is an electronic self-administered publicly available questionnaire. It is also available via the American Academy of Ophthalmology (AAO)'s IRIS patient portal, making it easy to incorporate into clinical practice in addition to valuation of medical devices.

In 2021, the AAO submitted PROWL SS for qualifications as a medical device development tool, or MDDT. Qualification is FDA's conclusion that within the context of use, MDDT has a specific interpretation and application in a regulatory review. Qualified tools minimize uncertainty in regulatory review for industry, reduce resource expenditure, and foster innovation.

PROWL SS includes 32 items from the full-PROWL questionnaire and assesses four key visual symptoms and patient satisfaction with vision. PROWL SS is the first ophthalmic MDDT qualified by FDA. PROWL SS is specific for LASIK population. However, tools developed in one setting can be modified or adapted for use in other contexts of use through bridging studies. PROWL is currently being used for other refractive surgeries in this manner.

Recently, we completed development of PRO for premium IOLs. The need for this questionnaire was first identified during the 2014 FDA workshop on novel endpoints for premium IOLs. Subsequently, a task force was formed that generated several consensus statements and an advisory committee was established for PRO development in a unique collaboration between FDA, AAO, and industry. Assessment of intraocular lens implant symptoms instrument was developed as a result of this unique collaboration. The instrument assesses frequency and level of symptoms farther for 15 symptoms. Like PROWL, this instrument incorporates images and definitions to facilitate reporting of complex visual symptoms. Once published, this tool will be added to the IRIS patient portal and become available for use by surgeons in clinical practice.

In the glaucoma space, we have lead bi-coastal centers of excellence in regulatory science and innovation collaboration. We partnered with John Hopkins to determine patient preferences in glaucoma treatment. At the UCSF Stanford CERSI, our goal was to develop a PRO measure to assess health-related quality of life in patients with mild to moderate glaucoma. Development of this PRO had many phases and took a number of years. We just completed this journey and are analyzing the data. Once published, this PRO will join PROWL and premium IOL PROs in the IRIS patient portal.

With the completion of instruments for refractive, IOL and glaucoma spaces, the FDA is turning our attention patients with profound vision loss. We obtained funding and started initial qualitative development towards a PRO for this patient population. During our workshop on expediting innovation by electronic implants for vision restoration on October 24 and 25, 2022 we heard directly from patients about what matters to them. We will discuss further the most impactful ways to incorporate their voices in device innovation in this space.

Although we have made tremendous progress in development and implementation of PROs in ophthalmology, there is still a lot of work do. We applaud the MTM Vision for hosting today's workshop and for exploring best ways to incorporate the patient voice in clinical endpoints for diabetic retinopathy. Continuous collaborative efforts will move us towards patient-centric health care.

## Jennifer Sun

The DRD staging update is the first plank in several phases of the work to follow. The DRD staging project arose out of a growing realization that although the DRSS and similar scales, like the International Classification Scale, have been very useful for many decades to stage retinopathy and risk stratify patients, they have very important limitations. For one, they only address the vascular components of retinal disease, as was remarked upon by Dr. Dolly Chang. However, they only look at the central 90° of the retina,^3^ and we have the ability now to image out to 200°. The Diabetic Retinopathy Clinical Research Retina Network Protocol AA did not find an association with predominantly peripheral lesions on the color images, but did find an association of predominantly peripheral lesions on ultrawide field fluorescein angiograms with future risk of diabetic retinopathy worsening, independent of baseline patient DR severity.

These findings need to be incorporated into future systems. Fluorescein angiography, particularly ultrawide field images and OCT angiography images, allow us to document nonperfusion of the retina and potential areas of retinal ischemia in ways that have not been previously accessible. Although OCT central subfield thickness is not a registerable endpoint because of its very modest correlation with visual acuity outcomes, we use OCTs on a daily basis to decide when and how to follow to treat and patients. Many morphologic changes on these images of the neural retina that may be better correlated with visual acuity than thickness measurements.

At the start of the DRD staging project, our vision was that at diabetes onset we would start thinking about a multidimensional holistic scale that incorporates measures of worsening retinal vascular function with retinal neural function in the context of the biochemical milieu of the retina, and within the greater context of the patient's systemic health. We need to understand both how these aspects impact patients’ visual function, and how they take into account and impact patients’ quality of life.

Efforts over the last year have benefitted from incredible collaboration from six working groups that explore specific areas of focus in vascular retina, neural retina, basic and cellular mechanisms, systemic health, quality of life, and visual function. The leaders of these working groups have led regular meetings with brainstorming and consensus by teams of international experts. They've conducted targeted reviews of the literature based on their discussions with completion of a very granular and standardized evidence grades for each variable of interest. Manuscripts have been drafted, undergone independent external reviews, and the manuscript revisions for some groups are in final form and for others in near-final form. We have charged them to do two things. One is to identify priority variables that might be incorporated into that vision of the multidimensional graph that you saw. We also asked them to delineate key research gaps. A lot of work still needs to be done in this area, and a lot of validation needs to be performed for us to stand up new and updated staging systems.

Dr. Helen Colhoun's systemic health group did a fabulous job in a systematic review of risk factors for DRD and their risk factors did not quite fit the mold of the biomarkers that we look at. We asked groups to identify variables that were relevant to specific types of DRD in terms of subclinical DRD disease that was not clinically visible or evident, and early-stage, mid-stage, and late-stage clinical DRD. These categories are very general because while the DRSS allows us to pigeonhole early, mid, late-stage disease from a vascular standpoint, there were other options that we wanted the groups to incorporate as they felt necessary.

We asked the groups to parse which types of variables might be ready for current use or use within the next one to two years based on ongoing research. We also asked the groups to indicate those that were promising with unmet but clearly defined research needs that potentially could be accomplished within the next 5 years, and those that were potential and promising, but which had unmet research needs that would really need more than a 5-year window to accomplish.

The DRD staging project was focused on biomarkers of disease. We tried to fit these variables into a severity scale that could be used to stage disease for thresholds of treatment need and risk stratification. How does this relate to what we are doing today in clinically relevant endpoints, because there is a difference in this distinction. Biomarkers are characteristics that are evaluated or measured as indicators of natural history, disease progression, or treatment response. For clinically relevant endpoints we need to see which of these biomarkers can then be validated into surrogate endpoints. Surrogate endpoints are those that have reasonable likelihood to predict clinical benefit. Typically we do this through validating studies that use clinical endpoints that directly reflect how a patient feels, functions, or survives.

The breakout sessions discussed which endpoints might be clinical endpoints that directly reflect patient experience, and which ones might be surrogate endpoints that do not directly reflect patients’ feeling, functioning, or surviving but which could be correlated with this. Over time, we will also ask which of these surrogate and clinical endpoints might have potential to be brought into the registration space as primary endpoints for our pivotal trials. This kind of effort needs validation of endpoints for a very specific context of use in specific cohorts for specific indications.

The breakout groups were in four areas:

• Visual Function and Retinal Physiology, led by Adam Glassman
• Patient Reported Outcomes Measures, led by Stella Vujosevic
• Systemic Biochemical and Cellular Markers, led by Lloyd Paul Aiello
• Retinal Imaging, led by Jennifer Sun

This work will take 5 to 10 years, so we enlisted the input of future leaders in the space. Dr. Cindy Cai from Johns Hopkins worked with PROs measures, Dr. Roomasa Channa from Wisconsin worked with the retinal imaging group, Dr. Ward Fickweiler from Joslin will do systemic biochemical and cellular markers, and Dr. Tien-En Tan from Singapore worked for visual functioning and retinal physiology.

## Wiley Chambers: Regulatory Perspective on Endpoint Development

Dr. Chambers stated that the presentation reflected his views and not necessarily the views or policies of the FDA.

Endpoints often depend on trial design, and can be for safety, efficacy, or both. Visual acuity is often an efficacy and safety endpoint. The Food, Drug and Cosmetic Act states that safety and efficacy are determined using adequate and well-controlled investigations. A product must be shown to have the effect it purports under the conditions of use suggested in the labeling. The following are not acceptable as the sole basis for the approval: isolated case reports, random experience, reports lacking details, or uncontrolled studies or partially controlled studies.

Adequate and well-controlled trials must have a clear statement of the objectives, and the study design must permit a valid comparison. Subjects must have or be expected to get the condition. Bias must be minimized on the part of subjects, observers and analysts including in the assignment of subjects to groups in a balanced (randomized) manner. The method of assessment must be well defined and reliable, and the analysis of results adequate to assess the effects. In addition, the study design must permit a valid comparison with comparator arms by having similar dosing schedules and evaluation timepoints, and be meaningful to subjects.

The following may be acceptable endpoints for diabetic macular edema (either as a percentage of successful subjects or as a mean difference between groups):

1. Improvement in visual acuity by at least 0.3 logarithm of the minimum angle of resolution (either high or low contrast)
2. Prevention of loss of visual acuity by at least 0.3 logarithm of the minimum angle of resolution

Visual acuity is critically important to many individuals in the United States because it often becomes the determining factor for having a driver's license and the absence of a driver's license often equates to a loss of independence regardless of how well a patient assesses their own vision. However, central retinal thickness is not accepted as an endpoint because no correlation between visual acuity and retina thickness; and decreases in retinal thickness may be due to macular degeneration.

The following may be acceptable endpoints for diabetic retinopathy:

Improvement on the ETDRS scale by at least:

1. Three levels for systemically administered products, or
2. Three levels for bilaterally administered products, or
3. Two levels for unilaterally administered products, or

Prevention of loss on ETDRS scale by at least:

1. Three levels for systemically administered products, or
2. Three levels for bilaterally administered products, or
3. Two levels for unilaterally administered products.

Use of ETDRS scale is acceptable because the 10 year study data supports use in preventing vision loss. Prior to using alternative scales, data is needed to support that the alternative scale can do the same.

The following are not acceptable as endpoints:

1. Patients’ receipt of laser treatment (focal or panretinal)
2. Patients’ receipt of anti-VEGF treatment
3. Development of neovascularization (unless subject's baseline examination is more than three ETDRS steps away from a neovascularization level)

PROs are acceptable for comparing products which could allow differing routes of administration, efficacy and adverse events to be compared. PROs need to be fit for purpose, and the National Eye Institute Visual Function Questionnaire (NEI VFQ) is not a validated PRO. The FDA has provided a Guidance Document: “Patient-Reported Outcome Measures: Use in Medical Product Development to Support Labeling Claims” to help develop PROs.^15^

In responding to questions, Dr. Chambers noted that other endpoints are potentially acceptable if they predict visual function outcomes in the future. Scales including the ETDRS are expected to be sufficiently discriminatory such that at least a two-step change is needed on the scale to demonstrate a clinically significant change.

Another question asked if the FDA would commit to a particular endpoint being acceptable to support a regulatory submission. Dr. Chambers responded that the FDA permits many clinical protocols to proceed with endpoints that the Agency would not accept as supportive of efficacy, as long as the results of the protocol will advance clinical knowledge. If a commitment from the Agency that a particular protocol will support a regulatory submission is desired, than a “special protocol assessment” should be submitted. In its response to a special protocol assessment, the agency commits that the study design and endpoint will support a regulatory submission. This assurance commits the Agency regardless of any potential change in personnel at the Agency.

A participant asked for general characteristics that the FDA looks for to determine meaningfulness. Dr. Chambers responded that a meaningful endpoint is usually one that almost everyone agrees is important for “activities of daily living.” Everyone does not have to agree, but it is likely to be only a small minority that disagrees. Ultimately, a product is approved because the benefits outweigh the risks and since every product has risks, there has to be a definite benefit.

Dr. Chambers was asked that since the Agency does not accept laser treatment or any intervention as an endpoint, is there another alternative measure. Dr. Chambers suggested that endpoints can be based on anatomical changes that would prompt an intervention. Although there are often personal, socioeconomic, and other factors that lead to an intervention actually being performed, it is often possible to agree on specific anatomical changes as being an endpoint, and then a subject may meet the endpoint, regardless of whether they are treated or not.

## Kerstin Wickström

Regulatory perspective on endpoint development—an EU view. Dr. Wickström stated that the views expressed are personal and not necessarily the views of the European Medicines Agency (EMA), the Committee for Human Medicinal Products, or the Icelandic Medicines Agency.

What are the regulatory requirements on the validations of endpoints? The starting point is always to define the context of use. This is quite often rather vague but needs to be carefully detailed. How should the biomarker/surrogate endpoint be used? As a pharmacodynamic marker, an exploratory, secondary, or a primary endpoint? Should it be used for diagnostic purpose, or to enrich a study population? In which phase of development should it be used, and in which disease stage/severity?

If used in an exploratory setting for example to obtain the proof of concept or to aid in those selection, Dr. Wickström would be rather relaxed. However, then there is a risk of drawing the wrong conclusion. When intended to be used as a primary endpoint in a study for registrational purpose, the requirements are high and there is a need to establish the link to and the relevance for the clinical outcome. Dr. Wickström might be more relaxed if it is used as secondary or exploratory marker, but it would likely not be acceptable to support claims in the label, especially not the indication.

Briefly, the basic principles for validation include demonstrating content validity that represents aspects of relevance for the disease, and construct validity to distinguish healthy from diseased as well as the different severity stages. The endpoint should be reliable, and should be fit to detect a change when the disease improves or worsens, or be stable in stable disease. Ultimately, it must demonstrate that it changes as a response to intervention.

For a surrogate for the primary endpoint to be used in a registrational trial, it is thus not sufficient to demonstrate a correlation with an established outcome measure. The change the endpoint measures must be demonstrated to contribute to something that would translate into a relevant benefit for the patient. For example, if the progression of a retinal structural change is reduced a certain extent every year, what would that mean? If the structure improves, does that mean that function improves? Would an ophthalmologists tell the patient: ”You have some side effects of treatment, but your progression is reduced with 20%. Unfortunately, we do not have a clue whether part of your visual function will be spared and whether you will notice it.” Data are consequently needed to interpret and to decide on the clinical relevance of a treatment effect. This might not be easy, especially in slowly progressive conditions there are challenges and it might not be possible to establish the link to and the relevance between the marker and the clinical outcome within a reasonable timeframe.

To determine the meaningfulness of a change, comparing the marker against established outcome measures as well as with quality-of-life instruments would set the basis. Additional support might be obtained from natural disease history data, other biomarkers or anatomical markers. If all data consistently point in the same direction, the case is obviously stronger. Another way forward might be to enrich the patient population with those who likely progress faster to get a readout within a shorter timeframe, but this may affect a future indication. To support efficacy in the longer term and the meaningfulness of treatment, modeling approaches, for example as done in glaucoma, could be explored to estimate how many years of useful vision a patient is expected to have. At the end of the day there might still be remaining uncertainties and the question might well be whether a conclusion on the benefit versus the risk can be drawn.

It is recognized that even if exploring these options, there might be difficulties. Regulators are happy to provide scientific advice to discuss options. Besides the scientific advice procedures that are provided at EMA and European national agencies, at EMA there are two voluntary procedures to obtain specific input on the qualification of biomarkers and methods. First, the qualification advice where input on the plans for validation is provided. It is a confidential procedure, but if the sponsor agrees and if the regulators consider the approach being promising, a public letter of support could be issued. When there are sufficient data to support the validity of a marker, a sponsor might request a qualification opinion. This is issued by the Committee for Human Medicinal Products. If a positive opinion is adopted, it would mean that there is regulatory agreement that the marker is acceptable to use within its defined context of use.

## Ulrich Luhmann

Ulrich Luhmann was very glad to attend the meeting and expressed his enthusiasm to share some key considerations and lessons learned from visual function testing in clinical trials and natural history studies in intermediate age-related macular degeneration and DRD. He reminded himself and the audience about his hope that during this exciting symposium everyone would rally around the common goal of new clinical endpoint development and that by keeping this in mind during daily working and by a willingness for collaboration and sharing this big goal may be achievable.

**Figure 3.**
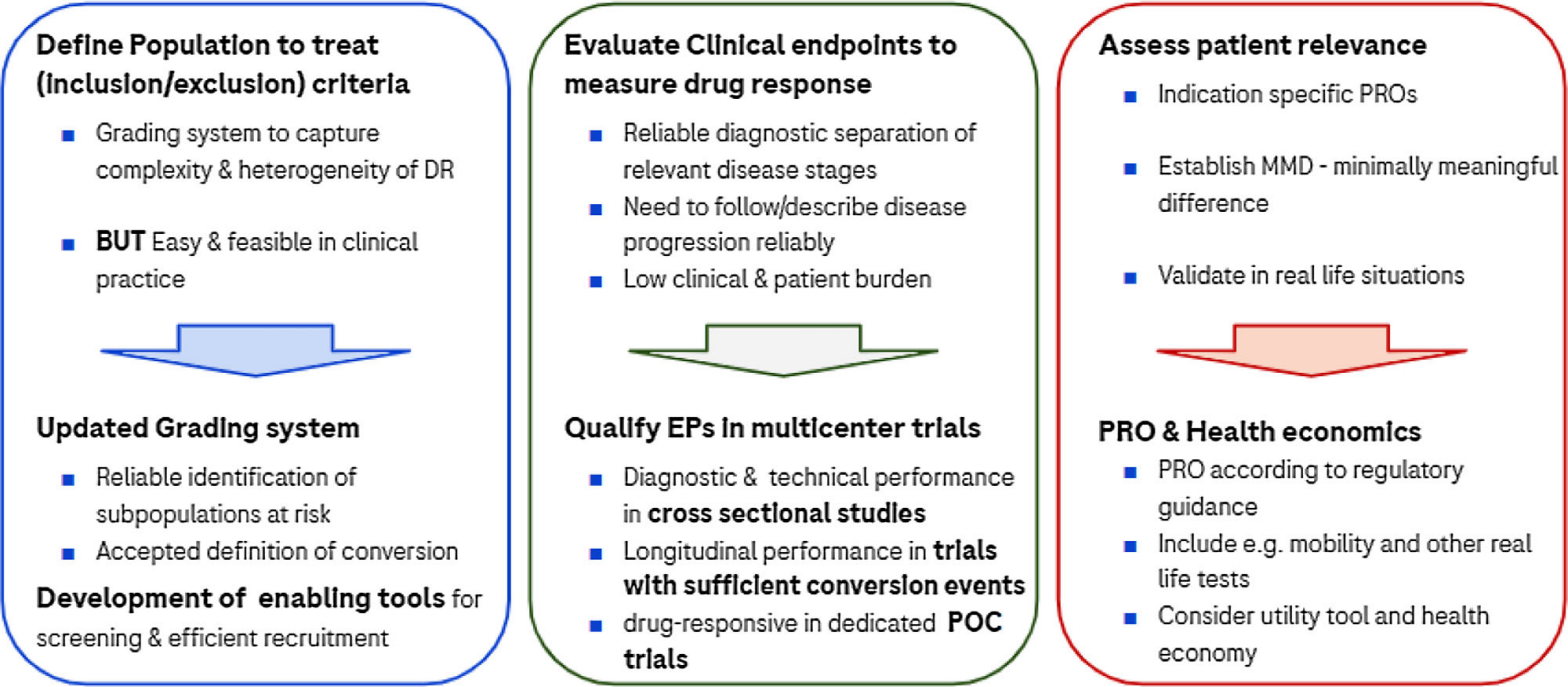
Three areas to address to enable drug development for NPDR.

The key focus area according to him should be to work on earlier disease stages, in particular in the nonproliferative stages of DRD, where a key need is to understand the clinical heterogeneity of the population. The main aim should be to identify patients who need treatment, either because they are close to developing sight-threating complications or are close to conversion to late proliferative stages of the disease. By focusing on the identification of this specific population, one would identify patients that would have a reasonably high chance to more immediately benefit from a clinically meaningful benefit delivered by such novel treatments (Fig. 3).

But what are the endpoints that should be looked at and would these actually describe changes that are meaningful for patients? Generating data in natural history studies will help to answer these questions and it will be possible to open up the earlier disease stages for drug development utilizing novel endpoints beyond DRSS for the delivery of novel treatments beyond anti-VEGFs (Fig. 3).

When thinking about who should be treated, the fact that cannot be ignored is that DRD is inherently slowly progressing disease. Therefore, the capabilities to run trials in that space are limited in terms of finances and time since trials need to be long and large. They also face the question whether the right patients are included under the current classification. Currently high failure rates of trials may be faced owing to the inclusion of a large proportion of patients with unclear or variable definitions of their disease stage.

What is currently being used as a classification? The good news is that DRSS is a disease staging system accepted by regulators and provides a basis for a primary endpoint that can be used in trials, since its predictiveness for vision impairment of patients has been established in long-term trials, and it works. But it only works for drugs that address the vasculopathy aspects of the disease, such as anti–VEGFs and still comes with many feasibility challenges. One is its inherent variability of plus/minus one step DRSS changes and the requirement to show a better drug response over existing proof of concepts of anti-VEGFs that have a large drug effect. Furthermore, because the DRSS is a structural endpoint, trial burden for patients, clinical sites and companies is increased owing to the fact that regulators request additional supportive evidence for patient relevance, which is not well defined, and thus needs to be explored by the implementation of many exploratory (functional) assessments at risk. This makes participation for patients and sites more burdensome and less attractive, but also puts the financial and operational bar higher for drug development companies to execute and invest in such trials.

To summarize, the aims of the joint effort should be therefore: define populations in need of treatment, work on a new updated disease staging system that not completely revolutionizes what we have, because it works, but that integrates additional information about the disease, and will provide more reliable and simpler staging system(s) for implementation that enables more efficient trials. It is definitively a long way to go, as the development of new endpoints beyond the current ones require a very systematic stepwise approach that leads to their validation, and the need to establish the understanding of their patient relevance.

It is furthermore important to talk about a potentially unpopular aspect. In order to make drug development attractive, all pharma companies are also interested in how payors and health economic systems value the benefit of any new treatment in this space that they may provide. Clearly, if the value is considered high, it makes drug development very attractive, but if the novel treatment does not add any value for the health economy, there is also no benefit and incentive to develop such a new drug. So what are the means to achieve the development of novel endpoints in a way that minimizes the risk for each partner and still provides sufficient incentive and “short term” benefits to make investing into this daunting task attractive? How could this be done?

One possible way forward are consortia that already have been mentioned earlier. One good example for a consortium that aims to achieve very similar things for intermediate age-related macular degeneration, is the MACUSTAR consortium.^16^ It is a European industry–academia consortium formed in the precompetitive space under the umbrella of the Innovative Medicine Initiative of the European Union, where pharma and academic consortium members work very effectively together to run a long clinical trial to generate necessary data. The study design considers cross-sectional and longitudinal data readouts for candidate endpoints, and take these forward into a three-year or longer longitudinal follow-up period within a multicenter clinical trial. The challenge remains whether and in what time frame enough events can be observed that allows linking earlier disease features and novel endpoints prospectively with disease stages with recognized clinically relevant vision impairments as well as the definition of the conversion events that are recognized by regulators and the whole field to anchor the novel data.

To evaluate the visual impairment in the structurally defined intermediate AMD stage, the MACUSTAR consortium has made an large effort and included eight functional vision tests into their trial protocol and developed a novel PRO specific for the earlier disease stages of AMD. They observed a heterogeneous impairment of visual function, in which around 70% of intermediate age-related macular degeneration patients show at least one “abnormal” visual function performance outside the 5th or 95th percentile of a normal control group. This suggests that the structurally defined disease grading is not well related to function as it highlights several types of functionally impaired sub-phenotypes within one structurally defined intermediate age-related macular degeneration patient group.

Similarly, at baseline of one of the ongoing phase 2 trials for an NPDR population of DRSS levels 47 to 53, a heterogeneous functional impairment across five different visual function assessments has been observed. Specifically, in approximately 70% of the included NPDR patients an extreme visual function in at least one of the assessments were detected and classes as abnormal, if the value was outside the 5th or 95th percentile of a normal control group. While this suggests, that in both intermediate age-related macular degeneration and NDPR approximately two-thirds of patients suffer from some degree of functional impairment already, it remains to be answered how meaningful to the patient these visual impairments are.

In this context, it is interesting to consider data from self-reported vision-related function burden questionnaires that are not accepted by regulators. Nevertheless, in this data set patients with severe NPDR self-report similar visual-related functional burden in their daily life as patients with later stages of the disease. Therefore, it remains very important to understand who the individual NPDR patients with patient relevant impairment are. Are they ones who suffer from one particular function deficit, or are they may be the ones that combine several of the visual function impairments from different domains of vision, such as low luminance, contrast sensitivity or visual field deficits? Further work is required to understand this clinically, but also needs complementary development work for visual function assessments to evaluate these visual functional deficits, somehow combined clinical assessment instead of the currently need to use five or more individual tests in the trials to collect this data. So the key lessons regarding understanding functional deficits in earlier disease stages are that it is currently feasible to perform and characterize heterogeneity of the functional deficits in clinical trials, but that this comes with a large cost and huge operational burden to patients, sites and developing companies.

Finally, in addition to better clinical characterization of the earlier disease stages to understand functional deficiencies and other disease features beyond vasculopathy of DRD and next to the generation of long term follow-up data for regulatory purposes, attention needs be paid to further operational challenges. First, novel ways of identifying and finding patients with earlier disease for the inclusion into trials need to be established. Secondly, clinical sites and patients need to be educated and put into a position to perform functional and other novel assessments in clinical trials that today are not usually part of routine clinical practice or even widely available.

Only if functional deficits and patient relevant visual impairments can be characterized and specifically assessed by novel endpoint and patient related outcome tools and thereby a more in depth structure-function and patients’ relevance evaluations becomes available to support regulatory approval, will a path forward towards more effective trials emerge, that will deliver novel treatment options for DRD that go beyond the current mode of actions and late disease stages.

## Martin Pellinat

Vision Tree met Dr. Levine 10 years ago when we built the JDRF Clinical Trials matching software and want to share our experiences of interoperability and launching electronic PROs in diabetes. Our core passion is quantifying the patient's voice.

We see PROs as central to research and to quality initiatives to set roles-based alerts around patient report outcomes to triage patient answers to early intervention and preventive care and also using sensor data. We certainly see PROs being incorporated into value-based care initiatives, and requirements around reimbursement, and using patient engagement as that requirement. That evolution happens where patient-oriented outcomes are becoming a standard of care, not just used for research.

Having started the company in 2003, in the last 12 to 24 months, thanks to the 21st Century Cures Act, has brought to the marketplace a technology called Smart On FHIR. Epic and Cerner have launched app marketplaces, which allows for third-party cloud-based apps to be launched out of the electronic health record. We can bring in an entire electronic PRO portfolio across all specialties of care and be able to use a portfolio l to validate against a new PRO assessment that might be designed, which, of course is what we are talking about here in the working groups.

In the field of diabetes, the SWEET Initiative in Europe resembles a lot of what happened here as far as working groups and consensus building around determining minimal data sets that are relevant to a research initiative, and the result was that over half a million patients were brought to the EU Parliament in Brussels, and there was public health decision making having these 12 countries all participate in this diabetes registry.

Using the SWEET example, coupled with other multicenter research initiatives, having interoperability, whether it is an EHR app or just a file upload, to be able to participate no matter where persons are located is important, and then being able to send that data to another central location, in this case this is Medidata Rave that we are doing with the NCI, is also just an important aspect when implementing an initiative like this.

At the MD Anderson Hospital in Houston they've collected more than 13,000 PRO forms through our cloud-based platform and are now curating that data. This is for head and neck cancer trials, and we are also launching the Epic app market app here. It is really possible to have interoperability and solutions that can bring your initiative into the real world.

## Adam Glassman

Mr. Glassman discussed considerations when selecting and validating clinical endpoints. Clinical endpoints directly measure how a patient feels, functions, or survives. A new clinical endpoint might not necessarily correlate with visual acuity, but could be measuring something different. Surrogate endpoints are a substitute for directly measuring a clinical endpoint. They're useful when clinical endpoints might take a long time to study. However, extensive evidence is needed from a trial showing that a surrogate can be a predictor or a correlate of a clinical endpoint. There are 4 aspects of a clinical measurement to understand before considering whether the measurement is an acceptable endpoint: (1) characteristics of measurement; (2) association with disease; (3) logistics; and (4) statistical considerations.

### Characteristics of the Measurement Tool

The properties of the measurement tool, including its accuracy and objectivity, need to be understood.

Several characteristics of the measurement tool are important: (1) reproducibility—test and retests, intersession, intergrader, depending on how subjective or objective the measure is; (2) understanding learning effects or fatigue effects; and (3) the measurement range, which is not necessarily just an aspect of the machine itself, but also could be based on the cohort under study. These can be assessed by repeated measures on the same eye at a single time point, covering a wide disease spectrum. For example, limited data from contrast sensitivity exist where only 43 participants with reproducibility data over a small range of contrast sensitivity measurements was available. Much more data is needed to begin to understand what some of the characteristics of contrast sensitivity are and specifically whether a change is beyond what we would expect from measurement variability.

Another reproducibility study was presented using OCT measurements from the Diabetic Retinopathy Clinical Research Network.^17^ In this study hundreds of replicate measures were obtained from Spectralis OCT, providing a good sense of what defines a real change on OCT. Based on the presented data, there is confidence that anything over a 25-micron change is real, likely outside of the range of expected variability. Macular thickness on OCT is an objective measure, so there is an even distribution around zero of the replicate measurements, indicating that there is not much of a learning or fatigue effect. A lot can be learned from obtaining replicate measurements cross sectionally, but it takes many participants to understand those characteristics.

### Association With Disease

Association with the disease is the next category of interest and includes the following topics: correlation with disease, sensitivity to change, and meaningfulness. The easiest to assess is correlation with disease, which is the correlation of the clinical endpoint with the disease stage or the duration. It is usually measured cross-sectionally at a single time point, and compared with disease duration, or another outcome of disease severity. As an example, looking cross-sectionally at contrast sensitivity, even with relatively small sample size across a spectrum of disease severity from no diabetes through several stages of diabetic retinopathy, sensitivities do worsen throughout the course of the disease process.

It becomes more complicated evaluating change over time. A longitudinal study needs to evaluate how the disease changes compared with how the endpoint changes. This factors back with understanding how to detect a real change, and with understanding the measurement itself and what constitutes a real change. The length of the study depends on the natural history of the disease, which needs to be part of the discussions on study development.

For example, there's limited data on the relationship between change in contrast sensitivity and change in DR severity. Data from RIDE and RISE in a population of diabetic macular edema participants showed a modest relationship between change in contrast sensitivity and change in disease severity. An example outside of diabetic retinopathy but within diabetes, is a relatively new outcome that has gained a lot of popularity—percent time in normal glucose range. As percent time in range is lower and lower, which is bad—there is an increase in the prevalence of retinopathy and microalbuminuria.

The final aspect of this group is clinically meaningfulness, which is really challenging. A clinical endpoint directly measures how a person feels, functions or survives, looking for clinically important differences in the individual and in the cohort, and really trying to understand if it is a change, not just is it beyond reproducibility, but does it actually impact how the person is experiencing life?

### Logistics

Logistics including device availability, complexity of testing, and standardization must be considered. The Diabetic Retinopathy Clinical Research Retina Network is conducting a study that includes Humphrey visual fields, which is a perfect example of challenges that can come up with logistics. Many retina practices in the Network do not have this machine, so availability and cost are an issue, and it takes a long time to measure. There are challenges depending on body type and age to obtain Humphrey visual fields. It is a major challenge within retina practices despite extensive glaucoma experiences to standardize methods with written procedures and identify skilled technicians particularly.

### Statistical Considerations

Defining the outcome, whether it is continuous or time to event and thinking about all the pros and cons to binary versus continuous outcomes will be critical.

Clinical trials will be needed for endpoint validation. Endpoints must be measurable, reproducible and associated with disease. The sooner endpoint assessment can be prioritized into studies the better off we will be.

## Lloyd Paul Aiello

Dr. Aiello noted that it was a daunting task to summarize the many remarkable events that transpired during the meeting. Given the fact that there were dozens of world experts in attendance with many divergent respective areas of expertise, he offered thoughts on the day's events.

Dr. Aiello noted that one of the key reasons for the gathering was a result of the extraordinary advances that had transpired in this field over a single patient's lifetime. He described the thousands of patients at Joslin who have had 50 or more years of type 1 diabetes, and several who had 80 or more years of type 1 diabetes. And yet, it was only 60 years ago that pituitary oblation was being used for treatment of diabetic retinopathy. It was only 50 years ago that the laser was starting out and anti-VEGF has been around only for 10 years as an approved therapy.

There had been a remarkable need for adjustment over a short period. Even considering only the anti-VEGF drugs that came into existence during the last 10 years, it was understandable how some endpoints had not kept up with the new and future needs of the rapidly evolving research. Such change necessitated reassessment of staging endpoints as was done during this meeting.

Dr. Aiello reminded the group that Drs. Eydelman, Wickström, and Chambers had set the stage clearly with regard to the personal impact of the disease, the need for new approaches for structural and functional endpoints, the usefulness of consortium, the emphasis on adding persons with diabetes into the process and regulatory issues.

Dr. Aiello then asked, “So what did we learn? What did we accomplish today?” First, he thought it remarkable to realize the diverse composition of the attendees which alone spoke to the importance and breadth of the mission. Attendees included leaders in government, academia, patient advocates, industry and others, all working together to move this field forward in a manner to best take advantage of the advances that had occurred, and those advances that that all hoped to achieve in the future. And all with an emphasis to do so in the safest way possible.

Dr. Aiello asked if the group had made progress in the areas noted by Dr. Levine as most important during the morning session. Was this a catalyst? The diversity of excellence present, together with the discussions that resulted, clearly had engendered much new thought, interaction, and novel ideas.

With regard to Dr. Levine's point that we cannot solve a problem that we have not defined, Dr. Aiello noted the ongoing staging project clearly addressed that directly. Out of the staging project various targets were generated and the group had started to specify new endpoint definitions and considerations.

Dr. Aiello stated that Robert's statement that you cannot find cures without studying the human condition was addressed in terms of a biorepository. The discussions of studies that would follow and how one would look at the clinical condition were articulated. Then, with a platform type of approach to these studies, linked with the biorepository and incorporating excellent characterization, wide breadth, and possibly useful biomarkers, the potential for advancement was high.

Finally, Dr. Levine had noted that one cannot judge success unless you know what to measure. To that Dr. Aiello added one must also know how to measure it, and what counts towards that measurement. The day's regulatory, academic and industry discussions helped illuminate those needs.

Dr. Aiello thought it most important to realize that each group was able to identify high impact targets. Thus, there are currently numerous identifiable targets worth investigating. Such study would be worthwhile even if the impact was still a ways down the road or if they addressed the capacity for definitive studies to determine if criteria for a registerable endpoint could be met.

Cleary, there was much yet to do individually and together, in terms of refining endpoints, and where and how their use would impact future study. Then Dr. Aiello asked “what is most needed now in order to assure further progress and success down the road?” First, he thought the field needed to determine how they can continue these efforts. In that regard, support of the MTM Vision Initiative and JDRF bode well for continued progress. There was also the need to know if there was a clearly perceived need for such efforts? From all groups present at the meeting, the reply was a resounding yes that there were still issues important for continued study.

Finally, Dr. Aiello asked, “Will we dedicate the efforts needed to this end?” Dr. Aiello stated that would mean there needs to be a commitment to working together, and devote the time, effort and money required to make it happen. Herein, Dr. Aiello noted, success relied on each of the participants.

For inspiration at this stage of so much inherent uncertainty, Dr. Aiello suggested we should think back to 1968 when the Airlie House Symposium, a similar gathering, was held in Virginia. There was diabetic eye disease, and the field needed to know what new things could be done for it. That was a group of folks that got together around an important need, and out of that came the DRS study, the ETDRS study, and validation of laser therapy that remains the standard of care to this day, more than 50 years later. Dr. Aiello pointed out that the initial studies were going to evaluate pituitary ablation, but there were a few present that noted there was this novel approach—laser photocoagulation—which might be studied instead. What resulted was the DRS study, and eventually one of the greatest advances in clinical care which has literally prevented visual loss for hundreds of millions of people around the world.

“So just consider what might come from today's work,” stated Dr. Aiello. It was his strong belief that the benefits were likely to be well worth the efforts that will be required on everyone's behalf to bring the identified goals to fruition.

Dr. Aiello expressed the gratitude of all present to JDRF, the MTM Vision Initiative, and the University of Michigan, for their wonderful support of these efforts. He also recognized the tireless leadership of Dr. Gardner and Dr. Sun that had resulted in remarkable progress, despite a potentially difficult, and widely diverse group of constituents.

Finally, Dr. Aiello noted that a very special debt of gratitude was owed to Dr. S. Robert Levine for his exceptional foresight, dedication, and true passion to facilitate efforts to preserve, restore, and protect vision of people with diabetes. Dr. Aiello stated that the future of diabetic eye care, and more importantly, the lives of all persons with diabetes, would likely be far brighter as a result of all these efforts, and that the group had just taken some of the first steps along this important journey.

## S. Robert Levine

Dr. Levine reinforced the importance of sticking to timelines. The energy in the room and the amount of intellectual power and other resource power assembled at the workshop can, with sustained effort, allow us to achieve our ultimate goals, as shown in Table 1.

**Table 1.** DRD Clinical Endpoints Workshop

***Next Steps***
• Invitation to join “Clinically Relevant Endpoints in DRD” Consortium to continue collaborative effort initiated in this Workshop.
• Identification of additional stakeholders for this collaborative effort.
• Set a timeline for regular follow-up virtual convenings of the Consortium.
• Establish Working Groups in specific areas (e.g., endpoint identification, clinical study design) to support the Consortium.
• Before end of Q4 2022, lock down the consensus prioritized “basket” of clinically relevant and potentially registerable endpoints that could be recommended for inclusion in ongoing DRD trials or new trials for further assessment, validation, and comparative analysis.
• By end of Q1, 2023, identify the first clinical study to be undertaken, together.
• Set timeline for first DRD study launch with target start date within the next 6 to 9 months.

Everyone should feel formally invited to join the “Clinically Relevant Endpoints in DRD Consortium” to collaborate on the topics discussed here.

Dr. Levine's final note was of an image of Mary tossing her hat in the air to represent her “You're going to make it after all” attitude. Diabetes stole Mary's joy because Mary in her heart was a dancer, and at the end she could not walk across the room safely, or read, or sustain her autonomy. So the hat in the air is a restoration of her joy because we found solutions to restoring vision and preventing visual loss in people with diabetes.

## Supplementary Material

Supplement 1
