# Supplementary material for: Report From the 2022 Mary Tyler Moore Vision Initiative Diabetic Retinal Disease Clinical Endpoints Workshop
Source: Transl Vis Sci Technol. 2023 Nov 28;12(11):33. doi: 10.1167/tvst.12.11.33 (PMC10691397; doi:10.1167/tvst.12.11.33)
Supplement: Supplement 1 [file tvst-12-11-33_s001.docx]

Supplemental Table 1. Organizations represented at the workshop

Adaptive Sensory Technology

American Academy of Ophthalmology

American Diabetes Association

Bayer

Boehringer-Ingelheim

Chronicles Health

Emory University

Excitant Therapeutics

FDA, EMA

Harkness Eye Institute, Columbia University

Intelligent Evaluation of Diabetic Retinopathy (EviRed)

IRCSS Multimedica

JAEB Center for Health Research

Janssen R&D

JDRF

John Curtin School of Medical Research

Joslin Diabetes Center

Kodiak Sciences

Konan Medical

Kyoto University

Labcorp Drug Development

Lions Eye Institute University of Western Australia

LKC Technologies

Moorfields Eye Hospital

NEI, NIDDK

Novartis

Novo Nordisk

Optos

Patient representatives

Perfuse Therapeutics

Queens University Belfast

Regeneron

Research to Prevent Blindness

Retina Consultants of Texas

Roche/Genentech

Singapore National Eye Center

The Glaucoma Foundation

Université Paris Cité

University of Barcelona

University of Iowa

University of Michigan

University of Pennsylvania

University of Utah

University of Wisconsin

Valo

Vision Tree Software

Wilmer Eye Institute
